# The Application of Ultraviolet Treatment to Prolong the Shelf Life of Chilled Beef

**DOI:** 10.3390/foods12122410

**Published:** 2023-06-19

**Authors:** Shuang Teng, Junlan Gan, Yu Chen, Liyuan Yang, Keping Ye

**Affiliations:** 1 College of Food Science and Technology, Nanjing Agricultural University, Nanjing 210095, China; 2 National Center of Meat Quality and Safety Control, Nanjing Agricultural University, Nanjing 210095, China; 3 Jiangsu Collaborative Innovation Center of Meat Production and Processing, Quality and Safety Control, Nanjing Agricultural University, Nanjing 210095, China

**Keywords:** meat irradiation, chilled beef, total bacterial count, quality, preservation

## Abstract

This study simulated the storage conditions of chilled beef at retail or at home, and the sterilization and preservation effects of short-time ultraviolet irradiation were studied. The conditions of different irradiation distances (6 cm, 9 cm, and 12 cm) and irradiation times (6 s, 10 s, and 14 s) of ultraviolet (UV) sterilization in chilled beef were optimized, so as to maximally reduce the initial bacterial count, but not affect the quality of the chilled beef. Then, the preservation effect on the chilled beef after the optimized UV sterilization treatment during 0 ± 0.2 °C storage was investigated. The results showed that UV irradiation with parameters of 6 cm and 14 s formed the optimal UV sterilization conditions for the chilled beef, maximally reducing the number of microorganisms by 0.8 log CFU/g without affecting lipid oxidation or color change. The 6 cm and 14 s UV sterilization treatment of the chilled beef was able to reduce the initial microbial count, control the bacterial growth, and delay the increase in the TVB-N values during storage. Compared with the control group, the total bacterial count decreased by 0.56–1.51 log CFU/g and the TVB-N value decreased by 0.20–5.02 mg N/100 g in the UV-treated group. It was found that the TBARS value of the UV treatment group increased during late storage; on days 9–15 of storage, the TBARS values of the treatment group were 0.063–0.12 mg MDA/kg higher than those of the control group. However, UV treatment had no adverse impact on the pH, color, or sensory quality of chilled beef. These results prove that UV treatment can effectively reduce the microbial count on the surface of beef and improve its microbial safety, thus maintaining the quality of beef and prolonging its shelf life. This study could provide a theoretical basis for the preservation technology of chilled beef in small-space storage equipment.

## 1. Introduction

The characteristics of chilled beef, such as soft and elastic texture, low juice loss, high nutritional value, and delicious taste, make it popular among consumers [1,2]. However, during slaughtering, cutting, processing, and transportation, slaughter tools, personnel, cutting equipment, air, and water lead to cross-contamination of the carcass; thus, the beef is easily contaminated by microorganisms, resulting in meat deterioration [3,4]. During the hanging of beef carcasses at temperatures between 0 and 4 °C for postmortem aging, the total bacterial count further increases because the temperature control conditions cannot completely inhibit the growth of microorganisms [5]. Microbial contamination and reproduction are the major factors causing beef spoilage, and chilled beef has a short shelf life in commerce due to microbiological activity. Therefore, reducing the initial microbial count is imperative to maintain the freshness of chilled meat and extend its shelf life.

Traditionally, ultraviolet (UV) light irradiation has been used for water, air, and surface sterilization [6]. UV light is an electromagnetic wave with a frequency between visible light and X-rays, ranging from 100 to 400 nm. It can be divided into different regions: long-wave UV-A (315 to 400 nm), medium-wave UV-B (280 to 315 nm), short-wave UV-C (200 to 280 nm), and vacuum UV (100 to 200 nm). UV-C is a short-wavelength UV light with high energy that can be absorbed by cellular RNA and DNA, damaging and/or destroying the bacterial structures required for growth and replication, resulting in the death of microorganisms [7,8]. UV-C light could be a source for the sterilization of food surfaces, which had been approved by the U.S. Food and Drug Administration (FDA) in 2000 [9].

Compared with thermal treatment or the application of antimicrobial compounds, UV irradiation is a very simple and safe sterilization process due to its ready availability and non-generation of potentially hazardous chemical residues [10,11]. As a physical, non-thermal technology, UV irradiation has a positive consumer image [12]. Therefore, UV has become a research focus in the field of food preservation in recent years [13,14]. Several studies have confirmed the effectiveness of UV irradiation for the surface decontamination of meat. Kim et al. [15] found that UV irradiation had an inhibitory effect on the growth of pathogenic microorganisms in Korean native beef. UV irradiation was performed at 4.5 mW/cm^2^ for 0, 5, 10, 15, and 20 min in their study, and the irradiation time was directly proportional to the inhibition effect. Yeh et al. [16] reported that the application of UV (254 nm) at 800 μW/cm^2^ for 30 s can be used to improve *Salmonella* control in ground beef, with individual applications of UV light reducing approximately 1 log CFU/g. In another study, cut pieces of food samples contaminated with *E. coli* or *S. aureus* were submitted to UV-C (254 nm) irradiation for 0, 1, 2, 3, 4, 5, and 10 min, and the results showed that the number of *E. coli* in beef was reduced by (1.0 ± 0.2) log_10_ CFU/mL after 5 min of exposure, and in chicken and pork, the number was reduced by (1.6 ± 0.7) log_10_ CFU/mL and (1.6 ± 0.4) log_10_ CFU/mL after 4 and 10 min of irradiation, respectively [17]. Holck et al. [18] studied the UV-C irradiation (0.0075–0.6 J/cm^2^ dose) in reducing the microbial loads in raw and smoked salmon; L. monocytogenes were reduced by 0.2 to 1.1 log on raw salmon and 0.7 to 1.3 log on smoked salmon; and the shelf-life was increased by up to 7 days and 14 days for raw and smoked salmon, respectively.

UV-C light inactivates microorganisms by damaging their nucleic acid, which absorbs UV light; nucleic acids are the strongest 253.7 nm light absorbers. A UV lamp emitting a wavelength of 254 nm is very close to the maximum absorption of nucleic acids; therefore, such UV lamps can be widely used in food sterilization and disinfection. Although several studies have employed UV irradiation to reduce bacteria on meat surfaces and improve microbial safety [19,20,21,22], a study pointed out that UV light at 254 nm can generate free radicals, which lead to damage to vitamins and proteins, the destruction of antioxidants, the oxidation of lipids, changes in color, and the formation of off-flavors and aromas, which all adversely affect the food, particularly when UV treatment is applied in high doses [12]. The quality of beef can be compromised after extensive UV treatment. The UV sterilization conditions of chilled beef require further investigation and optimization to avoid adverse effects and meet the needs for practical application. In addition, the effect of UV light on the quality of chilled beef on sale and the shelf life after UV treatment has rarely been studied. Therefore, the purpose of our study was to simulate the storage conditions of chilled beef at retail outlets or at home, and apply UV sterilization technology in small-space storage equipment. We investigate an effective and low-dose UV treatment to reduce the bacterial count on chilled beef without impairing the quality parameters, thus extending its shelf life. The specific objectives of this study were to (1) obtain optimal UV sterilization conditions for chilled beef in different UV combinations of irradiation distance and time, and (2) evaluate the impact of the optimal UV treatment on the quality parameters of chilled beef during storage, such as the total bacterial count, TVB-N value, TBARS value, pH, color, and sensory evaluation.

## 2. Materials and Methods

### 2.1. Sample Preparation

Chilled beef longissimus dorsi was purchased from local supermarkets. The meat samples were trimmed of visible connective and adipose tissue, cut parallel to the muscle into equal portions of 250 g (approximately 8 cm × 3 cm × 3 cm), and individually packaged in fresh-keeping bags (Polyethylene, 30 cm × 40 cm, 0.025 mm thickness, Suzhou Surong Plastic Products Co., Ltd., Suzhou, China).

A total of thirty samples were selected and randomly assigned to the control and nine UV treatment groups in the step of evaluation of UV sterilization conditions. A total of 42 samples were selected and randomly assigned to the control and treatment groups in the step of evaluation of preservation effect, the control group and the treatment group consisted of 21 beef samples each. Samples were stored in the refrigerator before use, and the temperature was 0 °C (±0.2 °C).

### 2.2. Optimization of UV Sterilization Conditions

The storage condition of meat sell at retail or storage at home were simulated in the study, and the research was carried out in a small space (in a refrigerator), so that 6–12 cm irradiation distances were chosen.

**Figure 1:**
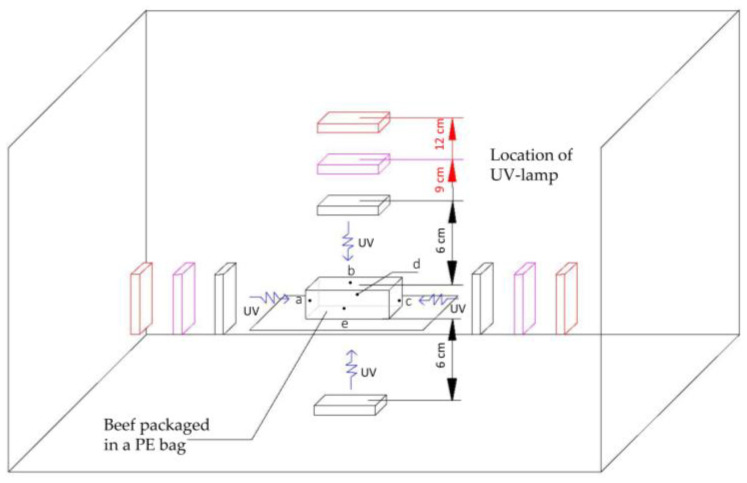
Schematic diagram of UV intensity test sites.

Nine groups of UV sterilization conditions were set up according to different irradiation distances (6 cm, 9 cm, and 12 cm) and irradiation times (6 s, 10 s, and 14 s), and the samples packaged in fresh-keeping bags without UV sterilization were used as the control group. UV treatment was carried out using the device shown in Figure 1. By measuring the total bacterial count, TBARS value, color, and sensory evaluation, the group with the best sterilization effect that did not affect the quality parameters was selected as the optimal UV sterilization conditions.

### 2.3. Evaluation of Preservation Effect

Chilled beef packaged in fresh-keeping bags was treated with the optimal UV sterilization parameters (UV at 6 cm for 14 s); the same batch of chilled beef without UV treatment was used as a control. The samples were stored at 0 °C (±0.2 °C) in a refrigerator; then, the total bacterial count, TVB-N value, TBARS value, color, pH value, and sensory evaluation were measured at 0, 3, 6, 9, 11, 13, and 15 days of storage.

### 2.4. Index Determination

#### 2.4.1. UV Intensity Detection

The parameters of the UV lamps were set as 254 nm wavelength, 150 mm length, and 5 W total power. The setting positions of the five UV intensity test points were shown in Figure 1. The UV intensity was determined by an ultraviolet radiation meter (UV-254, Beijing Shida Photoelectric Technology Co., Ltd., Beijing, China) when the distance between the UV lamps and the sample was 6 cm, 9 cm, and 12 cm.

Test point a is the left endpoint of the meat; test point b is the center point of the meat; test point c is the right endpoint of the meat; test point d is the endpoint of the meat near the inside of the box; and test point e is the endpoint of the meat near the outside of the box.

#### 2.4.2. Total Bacterial Count

The total bacterial count in chilled beef was determined according to the China National Food Safety Standard method GB 4789.2-2016 Food Microbiological Inspection Determination of aerobic plate count. Twenty-five grams of each sample was weighed and transferred to a homogeneous bag containing 225 mL of normal saline under aseptic conditions. After serial 10-fold dilution, 1 mL of the suspension was added to the plate, and PCA medium was poured in. After it was mixed and solidified, the number of bacteria was counted after incubation at 37 °C for 48 h. The results were expressed as the logarithmic number of colony forming units (CFUs).

#### 2.4.3. Sensory Evaluation

The standard for sensory evaluation of the freshness of the chilled beef was established according to the China National Food Safety Standard method GB 2707-2016 Fresh (Frozen) Livestock and Poultry Products. A 10-point scale (Table 1) was used for the assessment, with sensory parameters (color, odor, and texture) measured. Seven sensory experts were included in this study. The sensory assessors have good psychological quality and high sensory sensitivity. Their training includes meaning of the term and descriptive test. Three samples were presented at a time. Color, odor, and texture tested were through visual, taste, and tactile. Fresh meat samples were served as reference samples and presented to the sensory assessors at each storage period. Sensory evaluation was conducted in a room under controlled light, temperature (22–25 °C), and humidity. Samples were labeled with random numbers to avoid bias and evaluated within 15 min after opening the package. Each sensory assessment was conducted in the same setting without communication between members [23].

#### 2.4.4. Color [24]

Color parameters were measured using a colorimeter (CR-400, Konica Minolta, Tokyo, Japan). The aperture opening size was 8 mm, and the light source was D65. The observer angle used was perpendicular to the surface of the samples to obtain an accurate recording of the values. Five repetitions were performed for each sample, and color changes were described by L*, a*, and b*. The difference in color (∆E) between the UV-treated and control samples was calculated by the following equation:

$$\Delta E=\sqrt{\left(\Delta \;L\right)2\;+\left(\Delta \;a\right)2+\left(\Delta \;b\right)2}$$

∆L = L* − L_0_*, ∆a = a* − a_0_*, ∆b = b* − b_0_*. L*, a*, and b* are the measured values of the sample; L_0_ *, a_0_ * and b_0_ * are the measured values of the control group.

#### 2.4.5. Thiobarbituric Acid Reactive Substances (TBARS)

The TBARS determination of the chilled beef followed the procedure used by Erkan et al. [25] with some slight modifications. Five grams of ground beef was homogenized with 15 mL of 7.5% trichloroacetic acid (containing 0.1% EDTA). After filtration, 2 mL of collected supernatant was mixed with 0.02 mol/L thiobarbituric acid solution (2 mL) and boiled in water for 1 h. After cooling, the solution was centrifuged at 5000 rpm for 5 min at 4 °C. The absorbance of each sample was measured at 532 nm. Using a standard curve of tetraethoxypropane, the results were determined with malondialdehyde as the standard. TBARS value was expressed as the number of mg malondialdehyde (MDA) per kg of sample, which was calculated by the following equation:

$$X=\frac{c\times V\times 1000}{m\times 1000}$$

where *X* is the content of malondialdehyde in the sample (mg MDA/kg), *c* is the concentration of malondialdehyde in the sample solution obtained from the standard series curve (µg/mL), *V* is the volume of the sample solution (mL), and *m* is the mass of sample in the final sample solution (g).

#### 2.4.6. Total Volatile Basic Nitrogen (TVB-N)

Total volatile basic nitrogen (TVB-N) was measured according to the China National Food Safety Standard method GB 5009.228-2016 Determination of total volatile basic nitrogen in food. Five grams of the minced sample was dispersed in 50 mL distilled water for 30 min, then 10 mL of filtered supernatant was pipetted into the distillation tube. One gram of magnesium oxide was added to the distillation tube, and analysis was performed using a Kjeldahl automatic nitrogen analyzer (K1160, HaiNeng Instruments, Jinan, China). The results (*n* = 3) were expressed as mg of N per 100 g of sample.

##### 2.4.7. pH

The pH value in the samples was determined following the China National Food Safety Standard method GB 5009.327-2016 determination of pH value in food. The pH of each sample was measured in triplicate by inserting a pH meter (Model TESTO 205, TESTO Instrument Co., Ltd., Shenzhen, China) directly into the meat.

### 2.5. Statistical Analysis

The results were statistically analyzed using SAS 8.0 software and expressed as mean ± standard deviation (*n* = 3). The significance of the main effects was determined using the one-way ANOVA procedure, and the determination of significant differences (*p* < 0.05) among the means was performed by Duncan’s multiple-range and Tukey test. The graphs were generated using GraphPad Prime 8.

## 3. Results and Discussion

### 3.1. Evaluation of UV Sterilization Conditions

#### 3.1.1. UV Sterilization Intensity

The measurement of the intensity of UV radiation was taken inside the PE bag. As shown in Table 2, when the packaged chilled beef was exposed to the UV lamps, the UV light penetrated and acted on the surface of the meat. This phenomenon was consistent with the results of Tarek et al. [26] and McLeod et al. [27], who found that UV-C light can act on the sample surface through plastic films, such as polyethylene (PE) or oriented polypropylene (OPP). When the distance between the UV lamp and the sample was 6 cm, the UV intensity at test points a to e was significantly higher than that of other distance groups (*p* < 0.05). However, all test points obtained the lowest UV intensity when the distance between the UV lamp and the sample was 12 cm. When the irradiation distance was 6 cm, test point b received the largest area of exposure among all the test points, and the UV intensity detected at point b was the highest. Other points (a, c, d, and e) received a smaller radiation area, so the measured data were smaller than point b. As the irradiation distance increased, the transmission of UV light decreased, and the measured differences gradually decreased.

#### 3.1.2. Effect of UV Sterilization on Total Bacterial Count and Lipid Oxidation

**Figure 2:**
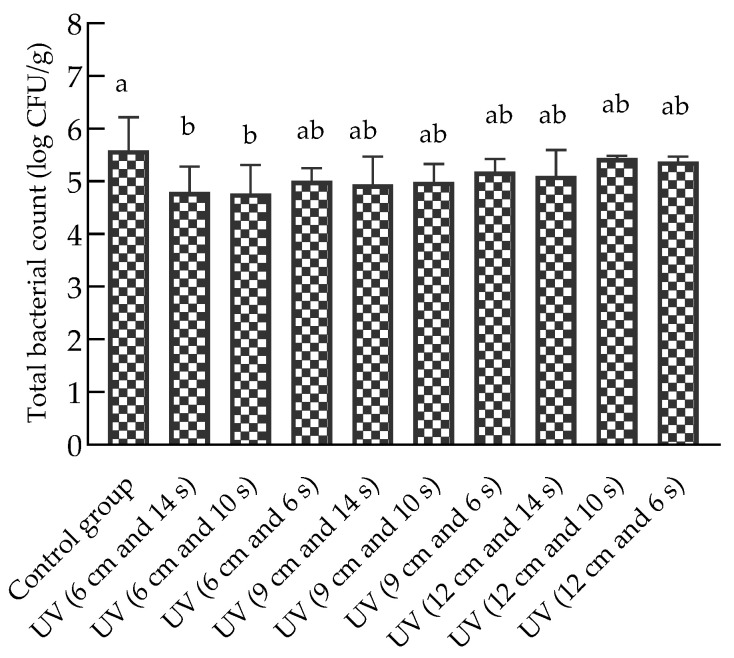
Effect of UV sterilization on the total bacterial count of chilled beef. Different lower-case letters indicate significant differences in total bacterial counts between the control and treatment groups (*p* < 0.05).

Figure 2 showed that the initial bacterial count of chilled beef without UV treatment was 5.59 ± 0.63 log CFU/g. The total bacterial count in the UV (6 cm and 14 s) and UV (6 cm and 10 s) treatment groups was significantly lower than that in the control group (*p* < 0.05); the total bacterial count was reduced by approximately 0.8 log CFU/g. Sobeli et al. [28] investigated the effect of pulsed light treatment at fluences of 0.525, 1.05, 2.1, and 4.2 J/cm^2^ on the aerobic mesophilic bacterial counts of beef loin steaks; the highest microbial inactivation of 3.49 ± 0.67 log CFU/g was determined under pulsed UV-C treatment at 4.2 J/cm^2^. Wang et al. [21] concluded that a larger area exposed to UV resulted in greater inactivation of microorganisms; microbial inactivation was proportional to the area of beef exposed to the UV treatment. Kalchayanand et al. [22] reported that when the distance between the surfaces of fresh beef tissues and the UVC source was 13 cm and exposure was 118 to 590 mJ/cm^2^, UVC treatment (254 nm wavelength) reduced aerobic bacterial counts by 0.64 to 1.00 log CFU/cm^2^. Although the samples used in our study varied, the chilled beef samples purchased from a market were used instead of inoculated fresh beef tissues and achieved a similar sterilization effect at a lower UV exposure dose.

**Figure 3:**
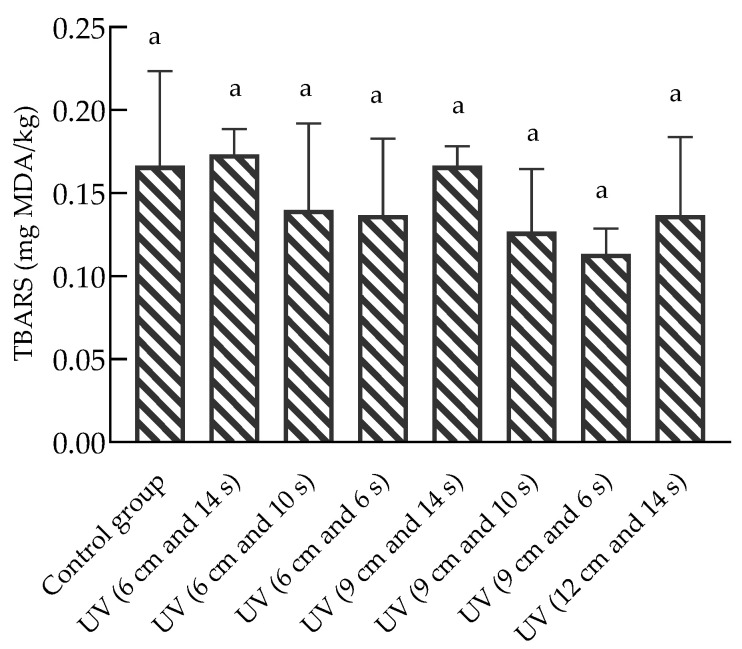
Effect of UV sterilization on TBARS values of chilled beef. Different lower-case letters indicate significant differences in TBARS values between the control and treatment groups (*p* < 0.05). The TBARS values of UV (12 cm and 10 s) and UV (12 cm and 6 s) were not tested.

The lipid oxidation of chilled beef in the different treatment groups is presented in Figure 3. The TBARS values of all test groups were between 0.12 and 0.17 mg MDA/kg, with the mean values of the UV (6 cm and 14 s) and UV (9 cm and 14 s) treatment groups slightly higher than those of the control group. The lowest TBARS value was detected in the treatment group with a UV irradiation time of 6 s. Park et al. [29] found there were 2.44- and 1.63-fold increases in the TBARS values (0.66 and 0.44 mg MDA/kg) of the chicken breasts irradiated with 3600 and 2400 mWs/cm^2^ of UV-C compared with the control (0.27 mg MDA/kg), so they pointed out that as the dose of UV-C increases, lipid oxidation gradually increases. In our study, under the same irradiation distance, the group with a longer treatment time showed higher mean TBARS values, while the group with a closer distance showed higher mean TBARS values within the same treatment time. However, the statistical results showed that no significant change was observed between each UV sterilization treatment group and the control group, indicating that UV treatment had no significant effect on the lipid oxidation of the packaged chilled beef. Lipid oxidation is a major cause of meat quality deterioration during processing and storage [30]. Some studies have reported initial TBARS values of beef longissimus dorsi of 1.4 [31] and 2.5 [5] mg MDA/kg. In this study, the results were similar to those of previous studies on TBARS values.

#### 3.1.3. Effect of UV Sterilization on Color

The color parameters of the UV-C light treated and untreated samples are shown in Table 3. There were no significant differences in the color (L*) of the chilled beef between the UV sterilized treatment groups and the control group. This result was supported by Sobeli et al. [28], who reported that the L* values of beef loin steaks treated with pulsed UV light were not significantly different compared to those of a control group. There were no significant differences in the color parameters a* and b* between the UV sterilized treatment groups and the control group.

The results of the total color difference showed that compared to the control group, the UV (12 cm and 14 s) treatment group obtained the smallest ∆E; in addition, ∆E in the UV (6 cm and 14 s), UV (9 cm and 6 s), UV (12 cm and 10 s), and UV (12 cm and 6 s) treatment groups were small.

#### 3.1.4. Effect of UV Sterilization on Sensory Evaluation

**Figure 4:**
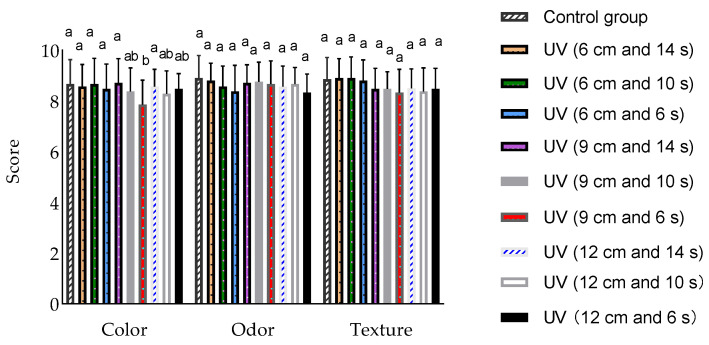
Effect of UV sterilization on sensory score of chilled beef. Different lower-case letters indicate significant differences in scores of color, odor, and texture between the control and treatment groups (*p* < 0.05). Determination of significant differences (*p* < 0.05) among the means was performed by Tukey test.

Figure 4 displays the sensory evaluation results (color, odor, and texture) of the control and treated group samples. Specifically, UV (9 cm and 6 s) scored significantly lower than the control group in color (*p* < 0.05). There were no significant differences between the nine UV treatment groups and the control group for odor and texture.

These results suggested that UV irradiation at a close distance (6 cm) and for a long period of time (14 s) was an effective method to reduce the initial bacterial count without affecting the lipid oxidation, color loss, or sensory evaluation of chilled beef packaged in a fresh-keeping bag. Therefore, the UV (6 cm and 14 s) treatment was selected to study the change in quality and freshness during storage in the following study.

### 3.2. Preservation Effect during Storage

#### 3.2.1. Total Bacterial Count

**Figure 5:**
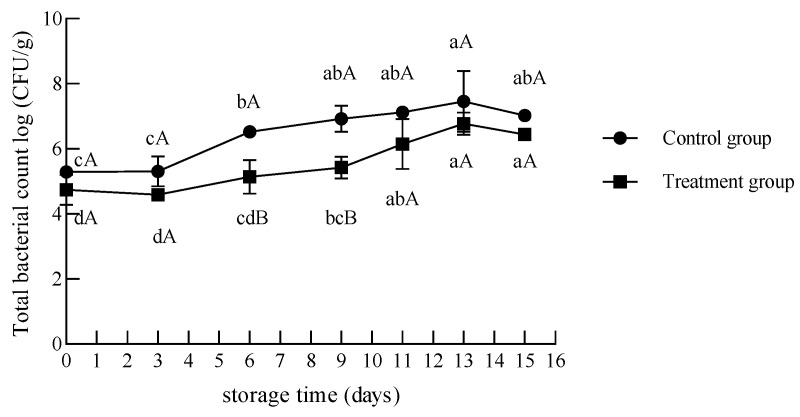
Changes in the total bacterial count of chilled beef during storage. Different lower-case letters indicate significant differences at different times with the same treatment, and different upper-case letters indicate significant differences with different treatment at the same time (*p* < 0.05).

The initial bacterial counts in the treatment and control groups were 4.73 ± 0.38 log CFU/g and 5.29 ± 0.16 log CFU/g, respectively. The changes in the total bacterial count of chilled beef during storage are shown in Figure 5. As the storage period increased, the total bacterial count tended to increase in the UV treatment group and the control group. In the UV treatment group, the growth of the total bacterial count from day 0 to day 6 was not significant, and the count at day 9 was significantly higher than those at days 0–3 (*p* < 0.05). The value increased to 6.14 log CFU/g at day 11, exceeding the acceptable threshold of 6.0 log CFU/g stated by the Chinese National Standard for spoilage. The total bacterial count in the control group significantly increased on day 6, as it reached 6.52 ± 0.11 log CFU/g, while that in the treatment group was 5.13 ± 0.43 log CFU/g. There were no significant differences in the total bacterial count between days 13 and 15 of storage, in spite of a slight reduction in the total bacterial count on day 15 of storage in the treated and control groups.

During days 0–11 of storage, the total bacterial count of chilled beef sterilized with UV-C was 0.56–1.51 log CFU/g lower than that of the control group. From days 6 to 9 of storage, the difference between the treated and control groups regarding the total bacterial count was more than 1 log CFU/g (*p* < 0.05), suggesting a slower growth rate than the control group.

These results indicate that UV sterilization treatment can reduce the initial bacterial count of chilled beef and inhibit the growth of the total bacterial count during storage. From the results of the total bacterial count, the shelf life of chilled beef in the treatment group was extended by 6 to 7 days. Lázaro et al. [32] also reported that a UV-C intensity of 1.95 mW/cm^2^ for 90 s promoted a decrease in the initial bacterial load and extended the shelf life of chicken breast meat stored at 4 °C.

#### 3.2.2. Total Volatile Basic Nitrogen (TVB-N)

**Figure 6:**
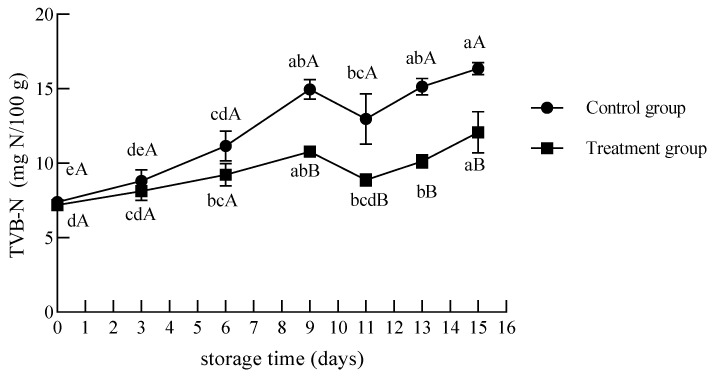
Changes in the TVB-N contents of chilled beef during storage. Different lower-case letters indicate significant differences at different times with the same treatment, and different upper-case letters indicate significant differences with different treatment at the same time (*p* < 0.05). Determination of significant differences (*p* < 0.05) among the means was performed by Tukey test.

The total volatile basic nitrogen (TVB-N) is generally considered the principal indicator for assessing the freshness and shelf life of meat [33]. As shown in Figure 6, the TVB-N concentrations of the UV treatment and control groups increased as storage progressed, ranging from 7.20 to 12.08 mg N/100 g and 7.40 to 16.36 mg N/100 g throughout storage. In fact, there were no significant differences in the content of TVB-N values between days 9, 11, and 13 of storage, in spite of the decreased mean TBV-N values of day 11 of storage, which was the same as that of the treatment group. Previously, Ishaq et al. [34] reported changes in the mean TVB-N value of raw beef from 10.34 to 29.48 mg N/100 g when irradiated with UV (distance = 8 cm; time = 60 s) before storage at 4 ± 0.5 °C for 15 days.

The TVB-N value of the UV treatment group was lower than that of the control group during storage, significantly from days 9 to 15 (*p* < 0.05). The TVB-N value of the UV treatment group was 12.08 ± 1.14 mg N/100 g at the end of the storage. The control group exceeded the acceptable limit of 15.0 mg N/100 g stated by China National Food Safety Standard GB 2707-2016 for fresh meat on day 13, suggesting rapid spoilage activities in the control group. TVB-N is produced by the degradation of proteins and other nitrogenous compounds caused by enzymatic degradation and microbial action in beef [35]. During storage, the TVB-N increased significantly with a significant increase in the total bacterial count, indicating that the increase in the TVB-N value is related to the increase in the total bacterial count. The primary mechanism of inactivation by UV was the creation of pyrimidine dimers, which prevent microorganisms from replicating, thereby rendering them inactive [12]. Therefore, UV treatment before storage reduced the initial microbial amount of chilled beef, repressed the elevation of the TVB-N value, and delayed the deterioration of meat. In addition, there was a lag in the time at which beef spoilage occurred, as judged by the TVB-N value in the China National standard.

##### 3.2.3. pH

**Figure 7:**
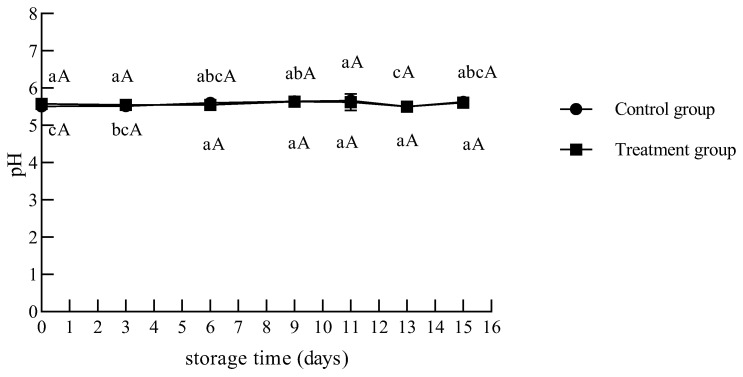
Changes in the pH value of chilled beef during storage. Different lower-case letters indicate significant differences at different times with the same treatment, and different upper-case letters indicate significant differences with different treatment at the same time (*p* < 0.05).

As shown in Figure 7, there were no significant differences in pH between the treated and control groups during storage. The pH of the chilled beef varied between 5.50 and 5.66 in the UV-treated and control groups. Reichel et al. [36] reported that the pH of pork during storage after UV treatment was between 5.29 and 5.37, which was slightly lower than that of the chilled beef, and there was no difference between the treated and untreated samples during 14 days of storage at 7 °C. Lázaro et al. [32] studied chicken breasts that were irradiated (0.62, 1.13, and 1.95 mW/cm^2^) and stored at 4 °C for 9 days, and reported that the UV-C-treated samples exhibited slight pH value variations during refrigerated storage. These studies supported the conclusion that UV-C light did not affect the important meat quality parameter of pH.

Moreover, some studies have shown that UV treatment can lead to changes in pH. Soro et al. [6] investigated the pH change in chicken breast fillets after LED-UV exposure at 280 nm for 6 and 10 min. At the end of storage (7 days at 4 °C), the pH of the control group was 6.50, while the pH range of the treatment group was 5.90 to 5.98, which was significantly lower than the control samples. They considered that changes in pH may be related to protein and lipid oxidation. Monteiro et al. [37] investigated the influence of UV-C doses 0.103 and 0.305 J/cm^2^ on pH in Nile tilapia fillets during 11 days of storage at 4 ± 1 °C. UV-C-treated groups demonstrated greater (*p* < 0.05) pH values than the control until the third day of storage, and lower pH values (*p* < 0.05) after the third day. They pointed out that it was due to the fact that UV-C light can promote protein degradation, which increases the amount of free amino acids, leading to a higher initial pH in the UV-C-treated samples. In our study, it was also found that the treatment group had a higher pH value than the control group during 3 days; however, there were no significant differences found by the statistical analysis.

#### 3.2.4. TBARS

**Figure 8:**
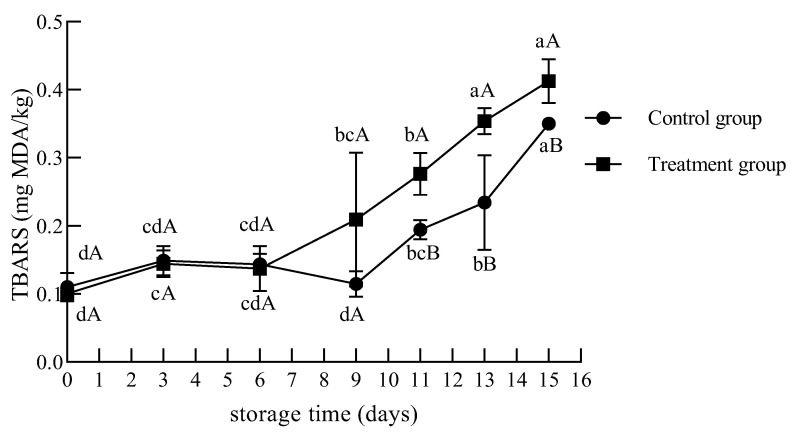
Changes in the TBARS contents of chilled beef during storage. Different lower-case letters indicate significant differences at different times with the same treatment, and different upper-case letters indicate significant differences with different treatment at the same time (*p* < 0.05).

As shown in Figure 8, the TBARS values increased from 0.10–0.11 to 0.35–0.41 mg MDA/kg during 0 °C storage in the UV-treated and control groups. The TBARS values changed little from days 0 to 6 and increased gradually from days 9 to 15. However, the TBARS value of the treatment group was significantly higher than that of the control group on day 11 (*p* < 0.05). This result indicated that the rate of lipid oxidation of chilled beef treated with UV increased in the late storage stage. Lipid oxidation products, such as aldehydes, have a negative effect on the odor and sensory shelf life of beef [38]. However, at the end of storage, the TBARS levels of the treatment and control groups were still lower than the fresh meat evaluation standard of 0.5 mg MDA/kg.

Chun et al. [19] measured the TBARS value of inoculated chicken breasts exposed to four different dose levels (0.5, 1, 3, and 5 kJ/m^2^) before storage at 4 ± 1 °C for 6 d; there were no significant differences in the TBARS values among all groups. Lázaro et al. [32] considered that UV-C irradiation did not affect the TBARS values, potentially because the exposure periods were insufficient to promote oxidation. Kalchayanand et al. [22] also did not detect significant differences in the TBARS values between UV-C-treated and control groups of fresh beef. However, this study found that the TBARS value of the UV treatment group was significantly higher than that of the control group on day 11 of storage.

#### 3.2.5. Color

**Figure 9:**
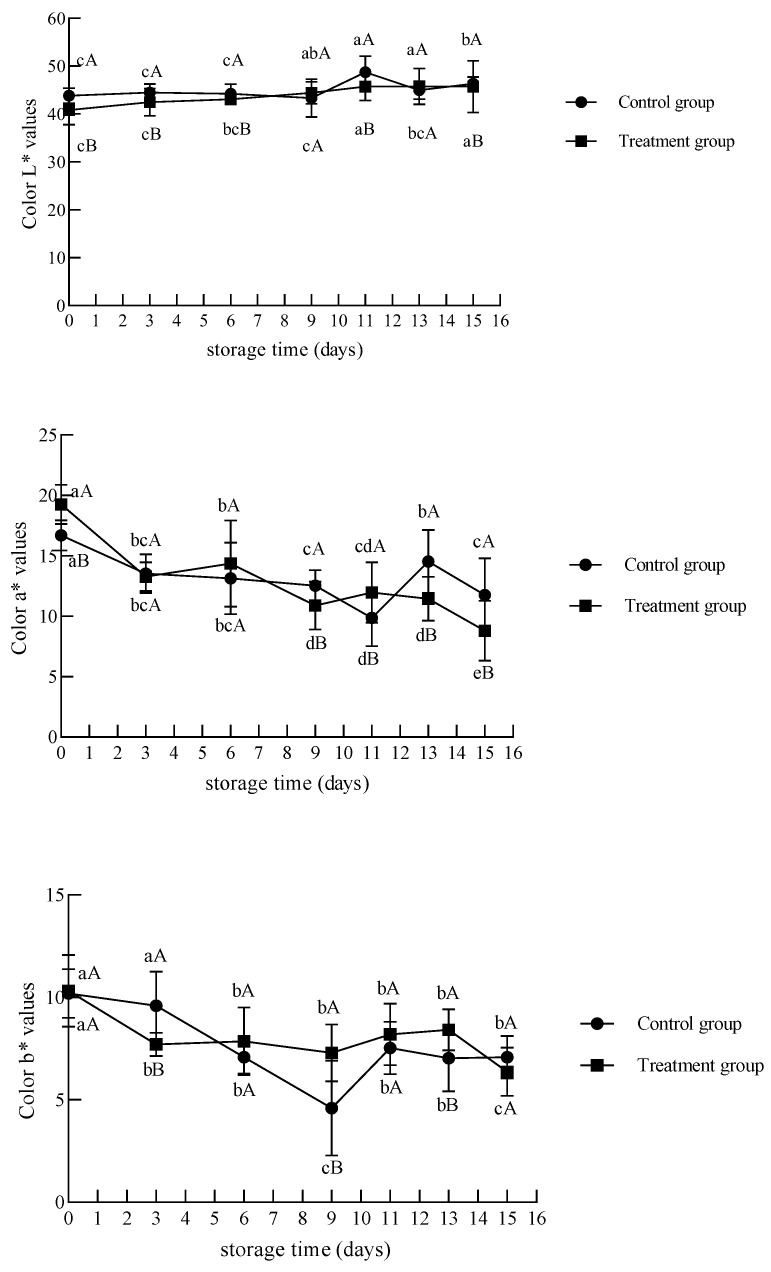
Changes in the color of chilled beef during storage. Different lower-case letters indicate significant differences at different times with the same treatment, and different upper-case letters indicate significant differences with different treatment at the same time (*p* < 0.05).

Color is an important element in evaluating meat quality [39]. The changes in the color (L*, a*, and b*) of beef at 0 °C during storage were shown in Figure 9. With an extended storage time, the L* values of the UV-treated group tended to increase. In general, as the storage time increased, the water retention of the meat decreased; water inside the meat seeped to the surface, which could improve the light reflectance and lead to an increase in L* [33]. L* values were significantly lower than those of the control group on days 0 to 6. Ishaq et al. [34] pointed out that the increase in the drip loss of muscles during storage was mainly attributed to the increased activity of microorganisms in the case of untreated samples. Due to UV irradiation before storage, the initial bacterial count of the samples was reduced, reducing the drip loss and improving the water retention capacity of meat during storage. A bright red color is usually identified with freshness and high quality [40]. With increasing durations, the a* values of the UV-treated group showed a decreasing trend, and there was also a decreasing trend in the a* values of the control group from day 0 to day 11. The b* value in the treatment group decreased after day 3 (*p* < 0.05) and changed insignificantly until day 13. In the early stage of storage, compared to the control group, the samples of the UV-treated group had significantly higher a* and lower L* values (*p* < 0.05), possibly because UV treatment changed the oxidation of myoglobin and the activity of microorganisms in meat. On days 9, 13, and 15 of storage, the a* value of the UV-C treatment group decreased significantly (*p* < 0.05), at which time the TBARS also showed a significant increase (Figure 7). It is possible that UV-C treatment engendered myoglobin and lipid oxidation, resulting in color loss [41].

Various studies have shown changes in the color values. Reichel et al. [36] irradiated pork with UV-C and did not find color alterations. Lázaro et al. [32] found a slight decrease in the a* values and a gradual decrease in the b* values during 4 °C storage; their results are consistent with our research. Chun et al. [19] also found decreasing a* values during 4 °C storage, and there was only a significant difference in the a* values of chicken breast on day 6. Lyon et al. [42] reported lower a* and higher b* values of chicken breast fillets on day 7 of storage when previously irradiated for 5 min at 1000 μW/cm^2^. However, the changes in color were considered minor and did not decrease the meat quality. In this study, it was confirmed that UV treatment did not have a deleterious effect on color.

#### 3.2.6. Sensory Evaluation

**Figure 10:**
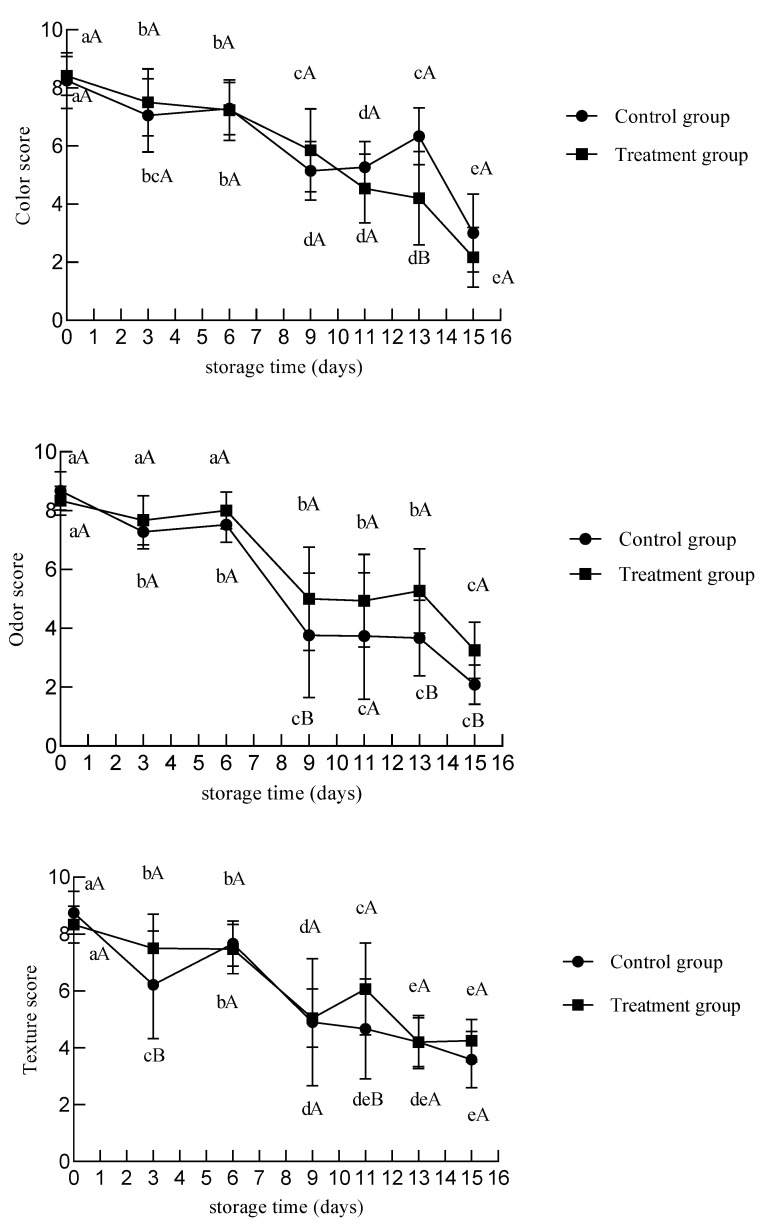
Changes in the sensory evaluation of chilled beef during storage. Different lower-case letters indicate significant differences at different times with the same treatment, and different upper-case letters indicate significant differences with different treatment at the same time (*p* < 0.05).

Sensory evaluation is the most direct way to evaluate the quality of chilled meat. Figure 10 showed that UV treatment had no adverse effect on the sensory scores of chilled beef during 0–6 days of storage. On the ninth day of storage, the odor score of the control group was lower than 4, expressing that the odor was not acceptable to consumers. On the 13th day, the sensory scores of the treatment group were 4.20, 5.27, and 4.20 in color, odor, and texture, respectively. On days 9–15 of storage, the odor and texture scores of the treatment group were higher than those of the control group, which may be attributed to the proliferation of microorganisms that led to the spoilage of the meat in the control group. Kim et al. [23] reported the effect of UV radiation (4.5 mW s/cm^2^) on the sensory evaluation of Korean native cattle beef. The odor did not change in the UV-treated and control groups during 4 days of storage at 4 °C, and decreased singnificantly until day 9. Within 4 days of storage, the control group showed greater tenderness decreases and succulence loss compared to the treated groups. Liu et al. [43] used pulsed UV light irradiation to treat traditional Chinese dry cured meat products, and found that the sensory score of the PL–UV irradiation group was 0.77 higher than that of control. This result indicated that PL–UV irradiation could improve the flavor. Similarly, our study also found that from the ninth day of storage, the UV treatment samples showed higher odor scores.

## 4. Conclusions

In this study, UV parameters of 6 cm and 14 s were selected as the optimal conditions for UV sterilization of chilled beef packaged in fresh-keeping bags. The results of the UV sterilization treatment before storage showed an inhibition of the total bacterial count and TVB-N content, and no adverse effects on the quality indicators of the chilled beef (pH, color, and sensory acceptability) during storage. Additionally, although fat oxidation accelerated during late storage, the TBARS value did not exceed 0.5 mg MDA/kg throughout storage. Hence, UV technology can effectively decrease the surface contamination of chilled beef, improve its safety, and extend its shelf life, reducing economic losses due to microbial contamination and the spoilage of meat. This study could provide a theoretical basis for the preservation technology of chilled beef, especially in refrigerators or other small-space storage equipment.

## Figures and Tables

**Table 1:** Sensory evaluation standard of chilled beef.

Score	Color	Odor	Texture
9–10	Bright red and shiny	Excellent	Soft and elastic, non-sticky surface
7–8	Red and shiny	Satisfactory	Good elasticity, non-sticky surface
5–6	Dark red and dull	Acceptable	General elasticity, slightly viscous surface
3–4	Dark or pale and dull	Poor	Inelastic, sticky surface
0–2	Dark brown	Very poor	Inelastic, strong surface adhesion

**Table 2:** Evaluation of the UV sterilization intensity of chilled beef.

Distance	Test Point a (W/m^2^)	Test Point b (W/m^2^)	Test Point c (W/m^2^)	Test Point d (W/m^2^)	Test Point e (W/m^2^)
6 cm	4.96 ± 0.03 ^aC^	5.14 ± 0.02 ^aA^	5.05 ± 0.02 ^aB^	4.40 ± 0.07 ^aE^	4.50 ± 0.03 ^aD^
9 cm	3.87 ± 0.01 ^bC^	4.00 ± 0.03 ^bA^	3.97 ± 0.03 ^bB^	2.95 ± 0.05 ^bE^	3.02 ± 0.04 ^bD^
12 cm	2.68 ± 0.01 ^cB^	2.68 ± 0.01 ^cB^	2.71 ± 0.01 ^cA^	1.95 ± 0.01 ^cC^	1.95 ± 0.01 ^cC^

Test point a is the left endpoint of the meat; test point b is the center point of the meat; test point c is the right endpoint of the meat; test point d is the endpoint of the meat near the inside of the box; and test point e is the endpoint of the meat near the outside of the box. Different lower-case letters indicate significant differences in UV sterilization intensity at the same test point for different distance groups; different upper-case letters indicate significant differences in UV sterilization intensity at different test points in the same distance group (*p* < 0.05).

**Table 3:** Effect of UV sterilization on the color of chilled beef.

UV-Group	Color L* Values	Color a* Values	Color b* Values	∆E
control group	41.97 ± 2.17 ^ab^	15.33 ± 1.87 ^a^	9.16 ± 1.04 ^a^	/
UV (6 cm and 14 s)	42.30 ± 2.76 ^ab^	15.05 ± 1.97 ^a^	9.33 ± 0.71 ^a^	2.46 ± 1.15 ^ab^
UV (6 cm and 10 s)	39.98 ± 2.77 ^b^	13.46 ± 2.06 ^a^	8.01 ± 1.32 ^a^	3.78 ± 0.50 ^a^
UV (6 cm and 6 s)	40.92 ± 3.28 ^ab^	13.50 ± 2.12 ^a^	8.65 ± 1.55 ^a^	3.74 ± 0.72 ^a^
UV (9 cm and 14 s)	40.56 ± 3.30 ^ab^	15.75 ± 3.10 ^a^	8.31 ± 1.27 ^a^	3.96 ± 0.68 ^a^
UV (9 cm and 10 s)	42.05 ± 3.16 ^ab^	13.98 ± 2.72 ^a^	8.01 ± 1.20 ^a^	3.70 ± 0.65 ^a^
UV (9 cm and 6 s)	43.75 ± 2.65 ^a^	14.90 ± 2.00 ^a^	9.56 ± 1.61 ^a^	2.71 ± 0.84 ^ab^
UV (12 cm and 14 s)	41.40 ± 1.99 ^ab^	16.09 ± 1.51 ^a^	9.29 ± 1.22 ^a^	1.27 ± 0.43 ^b^
UV (12 cm and 10 s)	43.37 ± 3.08 ^a^	14.53 ± 2.31 ^a^	8.44 ± 1.30 ^a^	2.96 ± 1.48 ^ab^
UV (12 cm and 6 s)	42.60 ± 1.07 ^ab^	14.34 ± 2.34 ^a^	8.00 ± 1.72 ^a^	2.31 ± 0.64 ^ab^

Data are presented as mean ± standard deviation, and different lower-case letters in each column indicate significant differences (*p* < 0.05). Determination of significant differences (*p* < 0.05) among the means (L*, a*, and b*) was performed by Tukey test.

## Data Availability

All available data are contained within the article.
